# Bilateral versus unilateral upper limb training in (sub)acute stroke: A systematic and meta-analysis

**DOI:** 10.4102/sajp.v80i1.1985

**Published:** 2024-01-23

**Authors:** Justine Dembele, Lisa Tedesco Triccas, Lisa Elogni Renaud Amanzonwé, Oyéné Kossi, Annemie Spooren

**Affiliations:** 1 REVAL, Faculty of Rehabilitation Sciences, Hasselt University, Diepenbeek, Belgium; 2 Department of Rehabilitation, National Reference Centre of Physical Medicine and Rehabilitation, Ouagadougou, Burkina Faso; 3 Unit of Neurology and NeuroRehabilitation, University Hospital of Parakou, Parakou, Benin; 4 ENATSE, National School of Public Health and Epidemiology, University of Parakou, Parakou, Benin

**Keywords:** bilateral training, functions, rehabilitation, (sub)acute stroke, upper limb

## Abstract

**Background:**

Integrating high dosage bilateral movements to improve upper limb (UL) recovery after stroke is a rehabilitation strategy that could potentially improve bimanual activities.

**Objectives:**

This study aims to compare the effects of bilateral with unilateral UL training on upper limb impairments and functional independence in (sub)acute stroke.

**Method:**

Five electronic databases (PubMed, Scopus, PEDro, ScienceDirect, Web of Science) were systematically searched from inception to June 2023. Randomised controlled trials comparing the effect of bilateral training to unilateral training in stroke survivors (< 6 months poststroke) were included. The treatment effect was computed by the standard mean differences (SMDs).

**Results:**

The review included 14 studies involving 706 participants. Bilateral training yielded a significant improvement on UL impairments measured by FMA-UE compared to unilateral training (SMD = 0.48; 95% CI: 0.08 to 0.88; *P* = 0.02). In addition, subgroup analysis based on the severity of UL impairments reported significant results in favour of bilateral UL training in improving UL impairments compared to unilateral training in “no motor capacity” patients (SMD = 0.66; 95% CI: 0.16 to 1.15; *P* = 0.009). Furthermore, a significant difference was observed in favour of bilateral UL training compared to unilateral UL training on daily activities measured by Functional Independence Measure (SMD = 0.45; 0.13 to 0.78; *P* = 0.006).

**Conclusion:**

Bilateral UL training was superior to unilateral training in improving impairments measured by FMA-UE and functional independence in daily activities measured by Functional Independence Measure in (sub)acute stroke.

**Clinical implications:**

Bilateral upper limb training promotes recovery of impairments and daily activities in (sub)acute phase of stroke.

## Introduction

Stroke remains the third-leading cause of death and disability combined in the world (Adoukonou et al. 2021; Feigin et al. 2022). Up to 80% of stroke survivors experience upper limb (UL) sensorimotor impairment at the (sub)acute stage, and few demonstrate complete functional recovery at 6 months post-stroke (Agbetou Houessou et al. 2021; Hayward et al., 2019; Kossi et al. 2016). The upper extremity is severely affected in 18% of cases (Persson et al. 2012), which leads to limitation in activities of daily living and reduction of quality of life (Sleimen-Malkoun et al. 2011).

Bilateral UL intervention after stroke involves practice of certain activities with both ULs to improve movement of the affected limb and includes both bilateral training (BT) with or without external assistance (Chen et al. 2019; Lee et al. 2017). Bilateral training includes repetitive practice of identical bilateral arm movements in symmetrical or alternating patterns and to bimanual training where both limbs perform different movements. A previous review and meta-analysis analysed the effect of BT compared to unilateral training on recovery of the UL after stroke (Chen et al. 2019). In a meta-analysis comparing the effects of bilateral and unilateral training, Lee et al. (2017) determined that constraint-induced movement therapy (CIMT) exercises were more effective than BT with regard to increased UL capacity (Lee et al. 2017).

At the neurophysiological and structural levels, the execution of bilateral movements post-stroke may facilitate cortical neural plasticity by these mechanisms: motor cortex disinhibition, increased recruitment of the ipsilateral pathways from the contralesional or contralateral hemisphere, upregulation of descending premotorneuron commands onto propriospinal neurons (Stinear et al. 2014) and interhemispheric interaction of affected and unaffected cerebral cortex (Latimer et al. 2010).

Due to neuroplasticity in the first 3 months after stroke (Stinear et al. 2020), this phase is therefore a critical window for experimental and restorative interventions to promote recovery after stroke (Overman & Carmichael 2014). Although previous meta-analyses compared the effect of BT and unilateral interventions, BT included various rehabilitation protocols, so BT alone has not been investigated separately. As a result, the previous analyses did not directly compare BT with unilateral training. To assess the effect of BT, it is important to compare different types of bilateral and unilateral training on the different levels of the International Classification of Functioning, Disability and Health (ICF), motor function levels, in both basic unilateral activities, and complex activities in which both hands are involved. Our study thus aimed to compare the effects of bilateral with unilateral UL training on UL impairments and functional independence in (sub)acute stroke, in a systematic review of the literature and meta-analysis.

## Methods

This systematic review and meta-analysis was performed according to our protocol, registered in the international prospective register of systematic reviews, PROSPERO (https://www.crd.york.ac.uk/prospero/logout.php; registration N° CRD42021251028). Our study was conducted according to the Preferred Reporting Items for Systematic Reviews and Meta-Analyses (PRISMA) guidelines.

### Data sources and literature search

Five electronic databases (PubMed, Scopus, PEDro, ScienceDirect and Web of Science) were searched for relevant articles published in English or French from their inception until December 2022. An update was made to extend search to June 2023. To initiate the search, general keywords were first designed using core concepts: population (stroke), intervention, comparator and outcomes. A more detailed search strategy using combinations of key terms related to core concepts and their synonyms was also carried out. The search strategy was adapted to each database with combinations of keywords and Medical Subject Headings (MeSH) terms used as applicable. Published reviews and the reference lists of retrieved publications were searched manually in databases.

### Study selection

After duplicates were removed, two reviewers independently examined the titles and abstracts of identified studies for relevance using EndNote X9 software. Full-text copies of potentially eligible studies were assessed and determined according to the following inclusion and exclusion criteria. Study selection was determined by consensus between reviewers, and rating was performed. Differences in scores were discussed until consensus was reached. When necessary, disagreements were resolved by consensus involving a third author.

The inclusion critera were randomised controlled trials (RCTs) published in English and French, involving acute and subacute (<6 months) stroke survivors (18) aged >18 years; investigating bilateral UL training like sensorimotor training, active and non-active movements; task-oriented training, strengthening and BT with or without a device, compared to unilateral training with or without a device; conventional therapy; neurodevelopmental therapy; conventional occupational or physiotherapy; electrical stimulation, to establish the effects of the interventions on UL function measured by the Fugl-Meyer Assessment for upper extremity (FMA-UE), the Wolf Motor Function Test (WMFT), Action Research Arm Test (ARAT), Box and Block Test (BBT) and the Functional Independence Measure (FIM).

Systematic reviews or meta-analyses, uncontrolled trials, clinical trials, quasi-randomised trials, case studies, stroke duration ≥ 6 months post-stroke, other neurological conditions apart from stroke and those with participants under 18 years of age were excluded. In addition, studies that did not provide data as mean scores and standard deviation (SD) of outcomes were excluded from the meta-analysis, and those not in line with the definition of the World Health Organization (WHO) pertaining to rehabilitation (WHO 2011) such as invasive and pharmacological interventions.

### Risk of bias assessment

Two authors used the Cochrane risk of bias tool to assess the risk of bias in studies. This tool assesses the risk of bias in seven areas: random sequence generation, allocation concealment, blinding of participants and personnel, blinding of outcome assessment, incomplete outcome data, selective reporting and any other bias (Higgins et al. 2011).

### Data extraction

Two types of BT were considered in our review. The first category was bilateral UL training with device-assisted BT and non-device-assisted BT. The second category was symmetrical or non-symmetrical bilateral UL training. In symmetrical training, both ULs perform identical movements to manage a task. In non-symmetrical BT or tasks functional bilateral training, both ULs perform typically functional tasks, e.g. closing a box.

The most common definitions reported for dose dimensions of motor intervention include the duration of practice reflected by time spent in a therapy, schedule of therapy, for example, frequency of sessions and intensity level of task (Dalton et al. 2022; Hayward et al. 2021). The included studies were also classified into two subgroups according to the training dose: (group 1) total duration of training ≥ 20 h or a session length ≥ 5 h per week considered as a high dose; (group 2) total duration of training of < 19 h or a session length < 5 h per week considered as a low dose.

Upper limb impairment severity in the included studies was assigned based on the FMA-UE scores or ARAT at baseline. Fugl-Meyer Assessment for upper extremity scores of 0–22 or ARAT scores of 0–10 represent no capacity; FMA-UE scores of 23–31 represent poor capacity and match ARAT scores of 11–21; FMA-UE scores of 32–47 represent limited capacity and match ARAT scores of 22–42; FMA-UE scores of 48–52 represent notable capacity and match ARAT scores of 43–54; FMA-UE scores of 53 through 66 or ARAT scores 55–57 represent full capacity (Hoonhorst et al. 2015).

### Data analysis

A meta-analysis was performed for the data synthesis using Review Manager Version 5.3 software, with a random effects model in which a *p*-value < 0.05 was considered significant. Effect size (ES) was estimated by calculating the standardised mean difference (SMD). The SMD reflects the intervention ES in each study relative to the variability observed in that study. An SMD of 0 means that the treatment and control have equivalent effects. Improvement is associated with higher scores on the outcome measure. Standardised mean differences >0 or <0 indicate the degree to which the treatment is more or less effective, respectively, compared to the control. Effect size was calculated based on means and standard deviations and on the sizes of the intervention and control groups. Heterogeneity was assessed using the results of the chi-squared test (significance level: *p* = 0.05) and the I^2^ statistic to quantify consistency. An I^2^ value of 50% or higher indicated the presence of substantial heterogeneity.

### Ethical considerations

This systematic review and meta-analysis did not require formal ethical clearance because all data were obtained from publicly available sources and were analysed anonymously.

## Results

### Study selection

**FIGURE 1:**
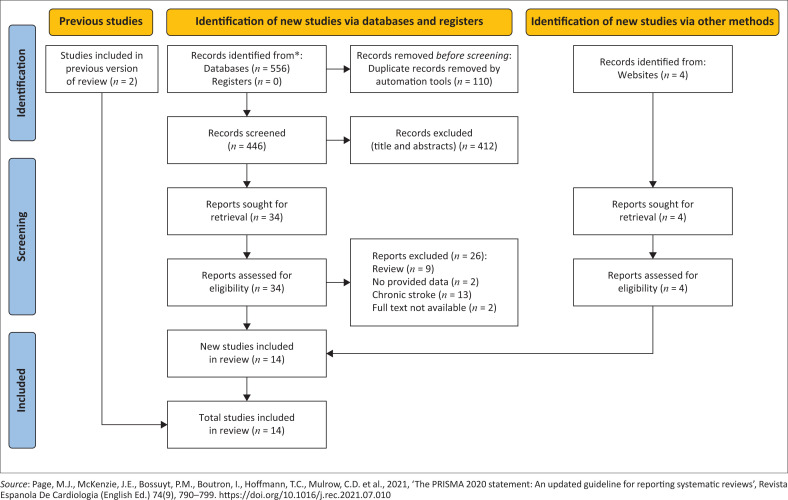
Study selection according to PRISMA Flow Diagram (Page et al., 2021).

Figure 1 shows an overview of our selection strategy process. A total of 558 studies were selected through electronic databases, while four additional records were identified through other bibliographic sources. After removal of duplicates, screening of titles and abstracts and reviewing of full texts, 15 RCTs met the inclusion criteria for the qualitative analysis and 14 studies were included in the meta-analysis. One trial was excluded for meta-analysis because data were not available either publicly or from the authors (Burgar et al. 2011).

### Study and participants characteristics

The main characteristics of the studies and participants in the 14 studies are shown in Table 1. A total of 706 participants were included with an age range from 49.3 to 74.3 years (SD from 2.0 to 13.22). Seven studies recruited patients with limited motor capacity with a FMA-UE score mean from 33 to 43 (Desrosiers et al. 2005; Dhakate & Bhattad 2020; Lum et al. 2006; Meng et al. 2017; Van Delden et al. 2015) or ARAT score mean 30 and 26 (Brunner, Skouen & Strand 2012; Easow & Chippala 2019); three other studies had participants with no motor capacity with a FMA-UE score mean from 6.6 to 17 (Hesse et al. 2005; Lee et al. 2017; Renner, Brendel & Hummelsheim 2020); in three studies, patients had poor motor capacity (Hsieh et al. 2017; Ma et al. 2022; Morris et al. 2008), and in one study, patients had notable capacity according to the aforementioned classification of severity above (Kumagai et al. 2022).

**TABLE 1:** Characteristics of the selected studies.

Author and year	Participants	Bilateral/bimanual UL training (BT)	Unilateral UL training (UT)	Duration (week)	Outcomes measures	Reported results	Follow-up
Brunner et al. (2012)	BT: N = 16 Age: 64.8 ± 12.8 years UT: N = 14 Age: 61.0 ± 10.0 years	Bimanual training based on daily life activities, strength, mobility training Dose: 4 h/week (16 h)	UT: mCIMT Dose: 4 h/week (16 h)	4	ARAT, 9-HPT, MAL	BT was as effective as modified constraint-induced movement therapy in improving arm motor function. No difference in change (*p* > 0.05) between the groups on any of the measures.	3 months No difference in change between the groups on any of the measures, neither at follow-up assessments.
Desrosiers et al. (2005)	BT: N = 20 Age: 72.2 ± 10.8 years UT: N = 21 Age: 74.3 ± 10.1 years	Symmetrical BT based on everyday tasks Dose: 45 min, 4 days/week (15 h)	UT based on functional activities and exercises Dose: 45 min, 4 days/week (15 h)	5	FMA, Martin Vigorimeter, Box and Block Test, Purdue Pegboard Test, FIM, AMPS	Analyses did not show any difference between the groups for the different dependent variables evaluated; arm impairments: *p* = 0.43–0.79; arm disabilities: *p* = 0.16, 0.90; and functional independence: *p* = 0.63 and 0.90.	NR
Dhakate et al. (2020)	BT: N = 20 Age: 54 ± 10 years UT: N = 20 Age: 54 ± 10 years	BT symmetrical based on functional activities Dose: 60 min/day, 5 days/week (20 h)	UT based on conventional physiotherapy Dose: 60 min/day, 5 days/week (20 h)	4	FMA-UE, FIM	BT proved to be more effective than conventional training programme to improve affected upper extremity motor function assessed using FMA (*p* = 0.002) and activity level assessed using FIM.	NR
Easow et al. (2019)	BT: N = 15 Age: 60 ± 9.35 years UT: N = 15 Age: 60.8 ± 9.9 years	Symmetrical BT based on proprioceptive neuromuscular facilitation Dose: 30 min/day, 6 days/week	UT based on proprioceptive neuromuscular facilitation Dose: 30 min/day, 6 days/week	NR	ARAT, FIM, NHPT	Difference between ARAT (*p* = 0.608) and NHPT (*p* = 0.787) from discharge to admission is not significant. Difference between FIM from discharge to admission is significant at *p* = 0.001.	NR
Hesse et al (2005)	BT: N = 22 Age: 65.4 ± 11.5 years UT: N = 22 Age: 64.0 ± 11.6 years	Symmetrical BT with computerised arm trainer Dose: 20 min/day; 5 days/week (10 h)	UT based on electrical stimulation Dose: 20 min/day, 5 days/week (10 h)	6	FMA, MRC, Modified Ashworth Scale	As expected, FMA score improved in both groups over time (F = 25.8; *p* < 0.001) but significantly more in the AT group. The UL motor power (MRC sum) significantly improved in both groups over time (F = 38.9; *p* < 0.001). The muscle tone remained constant in both groups.	3 months mean FM score was 13.5.1 Significant difference (*p* < 0.001) between groups in favour of AT. Mean MRC sum was (*p* < 0.001) higher in the AT group.
Hsieh et al. (2017)	BT: N = 16 Age: 49.28 ± 10.9 years UT: N = 15 Age: 52.87 ± 10.4 years	Symmetrical BT based on robotic priming with the task-oriented approach Dose: 90 min/day, 5 days/week (23 h)	UT based on tasks involved reach to grasp Dose: 90 min/day, 5 days/week (23 h)	4	FMA, Box and Block Test, grip strength, modified Rankin Scale, FIM, SIS	Primed group demonstrated significantly better improvement on the SIS strength subscale (*p* = 0.012) and a trend for greater improvement on the modified Rankin Scale (*p* = 0.065) than the unprimed group.	NR
Kumagai et al. (2022)	BT: N = 12 Age: 72.4 1 ± 7.6 years UT: N = 12 Age: 70.3 ± 12.1 years	Alternating BT using NHPT Dose: 10 trials of NHPT	UT performed only the task Dose: 10 trials of NHPT	1	NHPT, Purdue Pegboard Test, Box and Block Test	Improvement was comparable in both groups at post-training. Subanalysis: the alternating BT specifically for those with left hemiparesis.	7 days Not significantly different between the groups at follow-up.
Lee et al. (2019)	BT: N = 15 Age: 61.53 ± 8.81 years UT: N = 15 Age: 62.00 ± 8.13 years	Symmetrical BT based on task training Dose: 30 min/day, 5 days/week (10 h)	UT based on general rehabilitation Dose: 30 min/day, 5 days/week (10 h)	4	FMA	Results of BT were most significant. The mean change in FMA score was 4.27 ± 2.09 in the BT group and 1.80 ± 1.78 in the GULR group (*p* = 0.002).	NR
Lum et al. (2006)	BT: N = 10 Age: 62.3 ± 2.8 years UT1: N = 9 Age: 69.8 ± 4.0 years UT2: N = 6 Age: 59.9 ± 5.5 years	Symmetrical BT based on robot-bilateral (MIME) Dose: 1h/session, 15 sessions (15 h)	UT1 based on unilateral robot Dose: 1h/session, 15 sessions (15 h)	4	FMA, MRC, MSS, FIM, Ashworth Scale	Intervention group had advantages compared with CT producing larger improvements on FMA Scale. After treatment, a trend (*p* < 0.01) of greater gains was present in the robot groups compared with spontaneous recovery.	6 months – by the 6-month follow-up, FM scores in the robot groups exceeded those expected from spontaneous recovery (*p* < 0.05).
Ma et al. (2022)	BT: N = 10 Age: 59.00 ± 10.60 years UT: N = 9 Age: 56.44 ± 8.79 years	Robot-assisted task-oriented bimanual training Dose: 90 min, 5 days/week, for 4 weeks	UT based on conventional therapy Dose: 90 min, 5 days/week, for 4 weeks	4	FMA-UE, ARAT, WMFT	BT shows superior potential efficacy of the distal part of UL Significant improvement in the Fugl-Meyer Assessment (FMA and WMFT: *p* < 0.017). No significant improvements in ARAT.	
Meng et al. (2017)	BT: N = 64 Age: 55.38 ± 6.97 years UT: N = 64 Age: 55.19 ± 7.82 years	Hand-arm bimanual intensive training (HABIT) Dose: 2 h, 5 days/week (20 h)	UT-based conventional rehabilitation therapy Dose: 2h, 5 days/week (20 h)	2 weeks	FMA, ARAT, TMS (MPA, RMT, CMCT)	HABIT group showed improved scores compared to CRP group for FMA at *p* < 0.001, ARAT *p* = 0.022, AMP *p* < 0.001. However, CMCT at *p* = 0.054 and RMT at *p* = 0.088 were similar in groups.	NR
Morris et al. (2012)	BT: N = 56 Age: 67.9 ± 13.1 years UT: N = 50 Age: 67.8 ± 9.9 years	Symmetrical BT based on four core tasks Dose: 20 min/day, 5 weekdays (10 h)	UT Dose: 20 min, 5 days/week (10 h)	6 weeks	ARAT, 9-HPT	BT group demonstrated significantly greater change in dexterity (*p* = 0.03). There was no significant difference in change between groups at 0–6 weeks for ARAT scores (*p* > 0.05).	12 weeks – there was no significant difference in change between groups at 0–18 weeks (*p > 0*.05).
Renner et al. (2020)	BT: N = 35 Age: 62.68 ± 13.2 years UT: N = 34 Age: 61.05 ± 11.64 years	Symmetrical BT with cycle and spontaneous Dose: 60 min, 5 days/week (30 h)	UT Dose: 60 min, 5 days/week (30 h)	6 weeks	FMA, RRT, Modified Ashworth Scale	FMA score revealed a significant effect of time (*p* = 0.002) and no effect of intervention (*p* = 0.980). Elbow extension force increased significantly after BT Modified Ashworth Scale showed increased after BT compared to UT.	2 weeks
Van Delven et al. (2013)	BT: N = 19 Age: 62.6 ± 9.8 years UT1: N = 22 Age: 59.8 ± 13.8 years UT2: N = 19 Age: 56.9 ± 12.7 years	Symmetrical BT with Rhythmic Auditory Cueing (BATRAC) Dose: 60 min, 3 days/week (18 h)	UT1: mCIMT Dose: 60 min, 3 days/week Dose: 60 min, 3 days/week (18 h)	6 weeks	ARAT, FMA, 9-HPT, SIS	There were no significant between-group differences in change scores. However, the mBATRAC group showed greater movement harmonicity and larger amplitudes with the paretic hand after training than the mCIMT and DMCT groups.	6 weeks

mCIMT, modified constaint-induced movement therapy; BT, bilateral upper limb training; UT, unilateral upper limb training; UL, upper limb; ARAT, the action research arm test; MAL, motor activity log; MIME, mirror image movement enabler; FMA, Fugl-Meyer Assessment; FIM, Functional Independence Measure; WMFT, Wolf Motor Function Test; AMPS, Assessment of Motor and Process Skils; MRC, Medical Research Council, NDT, neurodevelopmental therapy; MSS, Motor Status Score; MPA, motor-evoked potential amplitude; RMT, resting motion threshold; CMCT, central motor conduction time scores; 9-HPT, Nine-Hole Peg Test; NIHSS, National Institutes of Health Stroke Scale; SIS, Stroke Impact Scale; ES, electrical stimulation; MSS, Motor Status Score; RRT, rate of rise of tension; TMS, transcranial magnetic stimulation; DMCT, dose-matched control treatment; CT, conventional therapy; BT, bilateral training; Hi, high; Lo, low; NR, not reported; FMA-UE, Fugl-Meyer Assessment for upper extremity.

Table 2 gives the type of BT described in each included study. Most studies were symmetrical biliteral training without device assistance.

**TABLE 2:** Types of bilateral upper limb training.

References	Device-assisted training	Non-device-assisted training	Symmetrical device-assisted training	Symmetrical non-device-assisted training	Non-symmetric bimanual training
Brunner et al. (2012)	-	✓	-	-	✓
Desrosiers et al. (2005)	-	✓	-	✓	-
Dhakate et al. (2020)	-	✓	-	✓	-
Easow et al. (2019)	-	✓	-	✓	-
Hesse et al. (2005)	✓	-	✓	-	-
Hsieh et al. (2017)	✓	-	✓	-	-
Kumagai et al. (2022)	-	✓	-	✓	-
Lee et al. (2019)	-	✓	-	✓	-
Lum et al. (2006)	✓	-	✓	-	-
Ma et al. (2022)	✓	-	✓	-	-
Meng et al. (2017)	-	✓	-	-	✓
Morris et al. (2012)	-	✓	-	✓	-
Renner et al. (2020)	✓	-	✓	-	-
Van Delven et al. (2013)	✓	-	✓	-	-
**Total**	**6**	**8**	**6**	**6**	**2**

Points indicate that the study has conducted the type of bilateral UL intervention. The first two columns group the bilateral training with or without device-assisted and the other 3 the symmetric or non-symmetrical bilateral training. Thus, all studies with device-assisted training are symmetrical. Non-device trainings are either symmetrical or non-symmetrical.

### Content and dosage of intervention

The details of interventions and the control groups are provided in Table 2. Six studies explored the effects of bilateral device-assisted training on UL motor function after stroke. The control group was unilateral UL training, including conventional training, for example, neurodevelopmental therapy. Eight studies investigated BT without device-assisted and these were functional tasks training. Among these eight studies, six studies performed symmetrical BT and two studies performed non-symmetrical BT.

The total duration of the interventions was 10–30 h of BT for 2–6 weeks while the duration of a session varied from 20 min to 2 h, 3–6 times per week.

### Risk of bias

Overall, 100% of studies presented low risk of bias with respect to random sequence generation, 64.29% with allocation and concealment, 14.29% with blinding of participants and personnel, 71.43% with blinding of outcome assessment, 28.57% with incomplete outcome data, 50% with selective reporting and 64.29% with other biases.

### Effectiveness of bilateral versus unilateral training on upper limb impairments

**FIGURE 2:**
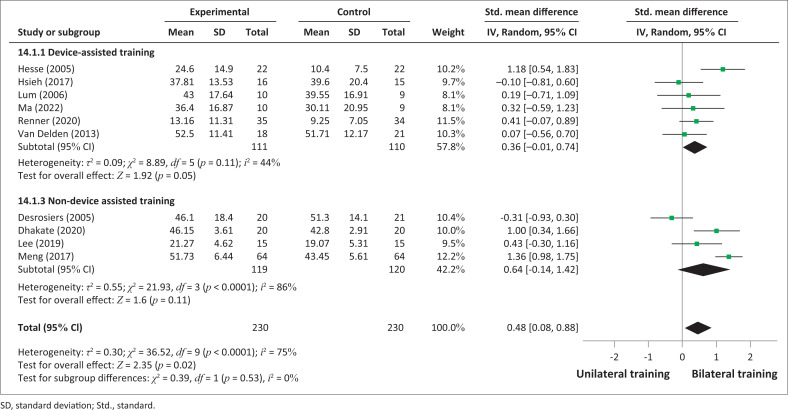
Comparative effectiveness of device or non-device-assisted bilateral training versus unilateral UL training on impairments (Fugl-Meyer Assessment for upper extremity) in subacute and acute stroke patients.

The results of the FMA-UE scores from 10 studies revealed a significantly improvement in favour of BT compared to the unilateral training group (SMD = 0.48; 95% CI: 0.08–0.88; *p* = 0.02) (Figure 2). However, high heterogeneity was present (I^2^ = 78%, *p* < 0.0001).

**FIGURE 3:**
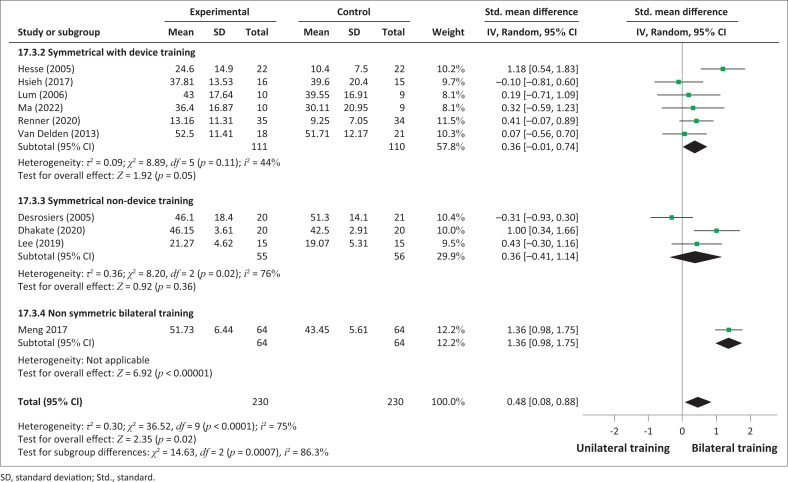
Comparative effectiveness of symmetrical or non-symmetrical bilateral training versus unilateral upper limb training on impairments (Fugl-Meyer Assessment for upper extremity) in subacute and acute stroke patients.

An analysis was performed according to the types of bilateral intervention on FMA-UE scores: device-assisted training and non-device-assisted training (Figure 2) and symmetrical with or without device training and non-symmetrical bimanual training (Figure 3). No significant improvements between bilateral UL training and unilateral UL training were observed in the analysis in terms of subgroups of interventions: bilateral device-assisted training (SMD = 0.36; 95% CI: −0.01 to 0.74) and bilateral non-device-assisted training (SMD = 0.64; 95% CI: −0.14 to 1.42). However, the results demonstrated significant improvement in favour of non-symmetrical BT (SMD = 1.36; 95% CI: −0.98, 1.75), but only one study was considered (Meng et al. 2017).

**FIGURE 4:**
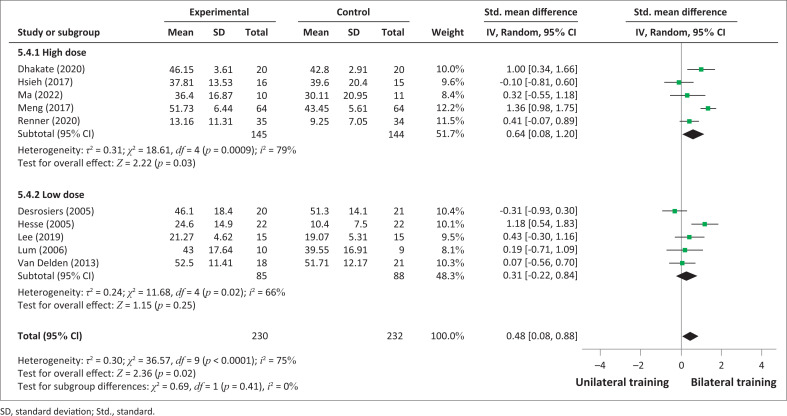
Comparative effectiveness of high-dose or low-dose bilateral training versus unilateral upper limb training on impairments (Fugl-Meyer Assessment for upper extremity) in subacute and acute stroke patients.

A subgroup analysis of high dose of FMA-UE scores showed significant effect (SMD = 0.64; 95% CI: 0.08–1.20; *p* = 0.03) but the low dose demonstrated no significant effect (SMD = 0.31; 95% CI: −0.22 to 0.85; *p* = 0.25) (Figure 4).

**FIGURE 5:**
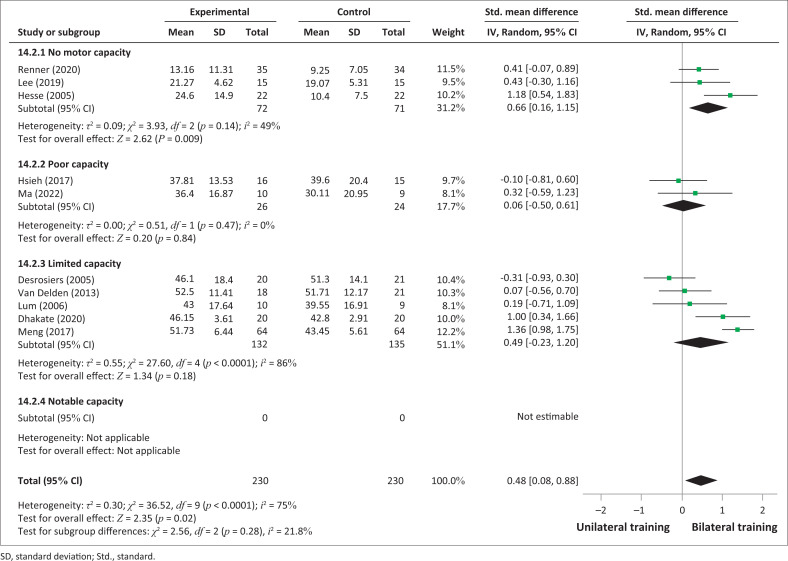
Comparative effectiveness of bilateral training versus unilateral upper limb training on impairments (Fugl-Meyer Assessment for upper extremity) in subacute and acute stroke patients according to severity of upper limb paresis.

Three trials included in the subgroup of ‘no motor capacity’ reported significant results in favour of bilateral UL training in improving the UL impairments compared to unilateral training (SMD = 0.66; 95% CI: 0.16–1.15; *p* = 0.009). Two studies (Hsieh et al. 2017; Ma et al. 2022) with ‘poor motor capacity’ participants showed non-significant results (SMD = 0.06; 95% CI: −0.50 to 0.61). Five trials included in the subgroup of ‘limited motor capacity’ have also demontrated no significant improvement (SMD = 0.49, 95% CI: −0.23 to 1.20). The comparative effectiveness on impairment (FMA- UE) according to the severity of UL paresis is represented in Figure 5.

### Effectiveness of bilateral versus unilateral training on upper limb activity limitations

The effects of bilateral UL training compared to unilateral UL training on activities analysed by the WMFT, ARAT and BBT scores of seven studies did not demonstrate significant improvement in overall effect of activities following a group of training (SMD = −0.09 points; 95% CI: −0.15 to 0.32). We observed homogeneity of studies (I^2^ = 34%, *p* = 0.17). The comparison revealed no significant difference in the analysis in terms of types of bilateral UL intervention compared to unilateral training. Based on dose of intervention and severity of UL paresis, the comparison between bilateral UL training and unilateral UL training showed no significant difference in the WMFT, ARAT and BBT scores.

**FIGURE 6:**
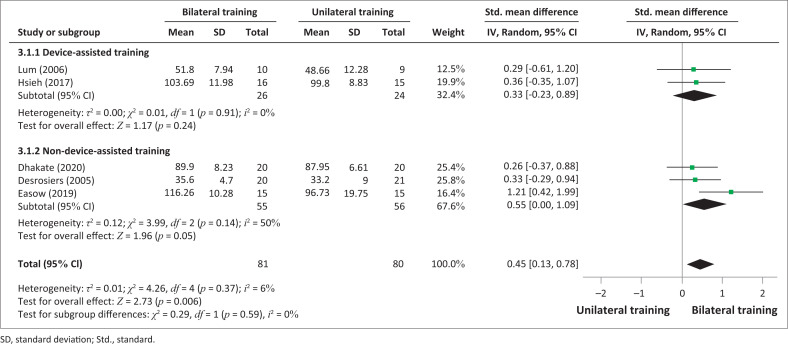
Comparative effectiveness of device-assisted or non-device-assisted bilateral training versus unilateral upper limb training on upper limb activities by Functional Independence Measure in subacute and acute stroke patients.

**FIGURE 7:**
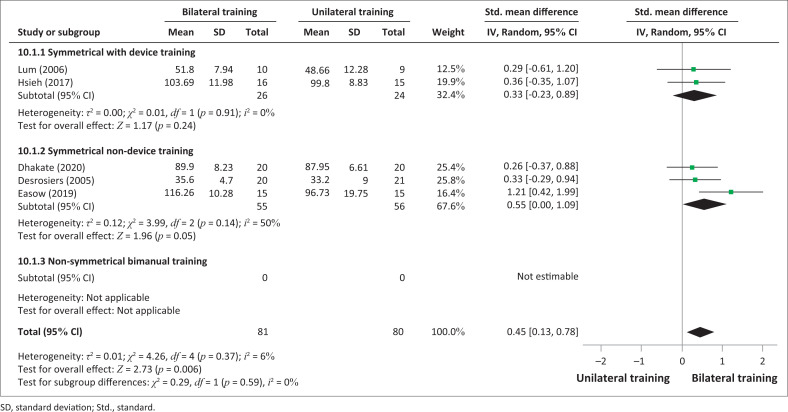
Comparative effectiveness of symmetrical or non-symetrical bilateral training versus unilateral upper limb training on upper limb activities by Functional Independence Measure in subacute and acute stroke patients.

However, a significant difference was observed in favour of bilateral UL training compared to unilateral UL training in terms of improvement of daily activities measured by FIM (SMD = 0.45; 0.13–0.78; *p* = 0.006) (Figures 6, 7). There was homogeneity in the studies (I^2^ = 6%, *p* = 0.37).

## Discussion

Our systematic review and meta-analysis aimed to compare the effectiveness of bilateral UL training with unilateral training on UL impairements and activities in acute and subacute stroke and to evaluate the influence of the types and dosage of BT and severity of UL paresis on UL recovery.

Our meta-analysis demonstrated that bilateral UL training is more effective than unilateral UL training in recovering of motor impairments measured by FMA-UE in (sub)acute stroke explored by 10 RCTs. Our results are similar to the results of Chen et al. (2019), who also reported significant improvement in overall FMA-UE scores in favour of BT in stroke patients (Chen et al. 2019). However, according to another meta-analysis, no significant differences were detected between BT and unilateral training for motor impairment (Chen et al. 2022). Currently, there are several types of UL BT in terms of content of intervention. Actually, all device training in our review was symmetrical, and we added an analysis of non-device training as symmetrical and non-symmetrical BT-based functional task training.

We have shown a significant improvement in UL impairment in favour of non-symmetrical BT compared to unilateral training. However, these findings should be interpreted with caution because the analysis was based on one study (Meng et al. 2017). Furthermore, the analysis shows that non-symmetrical training and training without technical assistance tend to have better improvement in UL activities measured by the WMFT, ARAT and BBT than unilateral training. As reported in other studies, repetitive bimanual movements can improve motor function promoting activity-dependent neuronal plasticity (Arya & Pandian 2014; Stinear et al. 2020). Regarding the positive effects of BT on motor function, several hypotheses have been proposed in the literature. Firstly, BT may promote positive neural interactions between sensorimotor areas in the ipsilesional and contralesional hemispheres to enhance coupling effects after stroke (Fan et al. 2015, 2016). Secondly, increased activity in sensorimotor areas after BT may contribute to functional reorganisation and neuroplasticity (McCombe Waller et al. 2014; Whitall et al. 2011). Thirdly, BT may restore normalised interhemispheric transcallosal inhibition (IHI) and reduce short-interval intracortical inhibition (SICI) in the ipsilesional hemisphere, both of which are associated with recovery of motor function after stroke (Swayne et al. 2008). Therefore, a higher dose of non-symmetrical BT could be further investigated so as to accurately highlight the effects on activities of daily living. In this field, more RCTs are needed to identify the optimal effect of BT based on functional tasks in UL activities post-stroke.

The significant improvement of motor impairments from BT was not accompanied by a greater improvement in UL activities as measured by the ARAT, WMFT and BBT compared to unilateral training. This is similar to two other systematic reviews (Chen et al. 2019; Coupar et al. 2010). Activity recovery is a very important goal for post-stroke patients in order to integrate the UL in daily activities (Nindorera et al. 2022). Daily activities sometimes require fine manipulation and some bimanual coordination. However, the majority of the included studies used analytical and symmetrical movements; in contrast, non-symmtrical bimanual movements with functional tasks were used less often. In addition, the lack of significant difference may be due to the outcome measures which were unimanual, yet the interventions were bilateral UL training.

Overall, as other studies did not report bimanual measures such as Adult Assisting Hand Assessment Stroke, it would be more appropriate that future interventions develop bilateral UL therapies focusing on functional tasks. In addition, they should include measuring tools that involve the use of both ULs, in a more natural situation in the activities of daily living. High scores on this tool can be obtained using only the nonparetic arm (Annabel 2018). However, the domains of self-care and transfers may require the use of both ULs. These two domains are composed of items based on activities of daily living performed by both ULs. Indeed, the items of this scale are based on bimanual activities of daily living in comparison with unilateral measures like WMFT and ARAT; thus, the practice of bimanual activities could have more impact in comparison with unilateral training. It is more likely that the greater improvement is due to the training of bimanual activities or bilateral activities. The need for more specific measures of bimanual activities is therefore necessary to support the conclusions of our review.

The dose of the training is important to reach functional recovery after stroke (Amanzonwé et al. 2023; Kossi et al. 2023; Nindorera et al. 2023). Interventions favouring intensive high repetitive task-specific training in all phases post-stroke have strong evidence for better results on motor function and activities (Veerbeek et al. 2014). With regard to subgroup analyses, our results showed that recovery of motor impairments can be favoured by intensive bilateral UL training in the acute and subacute stroke with at least 1 h of training, 5 times per week. Additional studies with bilateral task-oriented intervention, dimensions of dose articulation as proposed by Hayward et al. (2021) and long-term follow-up could provide more evidence on the effectiveness of motor function of the UL but are not reported. Chen et al. (2022) observed significant improvements in motor impairment in BT, when the dose of intervention was high.

Our analysis showed significant improvements for the subgroup of patients with ‘no motor capacity’ on pooled results of FMA-UE score for three studies. The effectiveness of bilateral UL training in patients with low motor capacity may be explained by the fact that patients use the two ULs and decrease intralateral inhibition in bilateral tasks when both hemispheres are activated (Stinear et al. 2020). The contribution of the healthy hand is therefore important in the management of patients after stroke (Van Gils et al. 2018). The healthy UL contributes to the movement of the injured side even with poor strength which allows a better recovery; however, in the unilateral UL training, it is difficult to do the training when the patient does not have a certain degree of motor strength. This training method could be further investigated in patients after acute and subacute stroke to support our results.

### Strengths and limitations

A strength of our systematic review and meta-analysis is that it includes only studies with high-quality scientific evidence, namely RCTs. In addition, to the best of our knowledge, our review may be the first meta-analysis looking at the effect of types of bilateral UL training in acute and subacute stroke. A limitation of our systematic review and meta-analysis is the heterogeneity encountered among the studies and limited studies in some analysed subgroups. In addition, the search strategy was limited to full publications in English or French; therefore, relevant publications in other languages may have been missed.

## Conclusion

Our systematic review and meta-analysis aimed to compare the effectiveness of bilateral with unilateral UL training in acute and subacute stroke. Our results noted that bilateral UL training was more effective in impairments, especially in the interventions with greater dose, severe impairment and on complex activities as measured by FIM. However, no significant effects were found on activities measured by ARAT, WMFT and BBT scores. The analysis of types of BT shows no significant difference between bilateral and unilateral training.
